# Productivity Improvements in Hip and Knee Surgery

**DOI:** 10.1155/2014/615784

**Published:** 2014-02-20

**Authors:** Frank A. Sloan, Linda K. George, Linyan Hu

**Affiliations:** ^1^Department of Economics, Duke University, P.O. Box 90097, Durham, NC 27708-0097, USA; ^2^Department of Sociology and Center for the Study of Aging, Duke University, P.O. Box 90088, Durham, NC 27708-0088, USA; ^3^Center for the Study of Aging, Duke University Medical Center, P.O. Box 3003, Durham, NC 27710, USA

## Abstract

Productivity improvements that occur as technologies become widely used are not well documented. This study measured secular trends over 1998–2010 in productivity of hip and knee procedures gauged in terms of changes in physical function and pain after versus before surgery. We used data from the Health and Retirement Study. Health outcomes from surgery were measured by 6 physical functioning scales and 2 pain indicators. We used propensity score matching to obtain nonsurgery control groups. Not only were there substantial improvements in physical functioning and pain reduction after receipt of these procedures in all years, but also we documented improvements in health outcomes over time. Largest improvements were for reductions in numbers of Activity and Instrumental Activity of Daily Living limitations for knee procedures.

## 1. Introduction

Technological change has led to substantial improvements in longevity and quality of life, but at the same time, a substantial amount of the growth of spending on personal health care services has been attributed to diffusion of new technologies [1–4]. A recent study concluded that technological change explained from 27 to 48 percent of the growth in spending on personal health care services in the USA that occurred between 1960 and 2007 [5]. While the introduction of breakthrough technologies receives much publicity, most technological change is incremental as are the vast majority of innovations following introduction of a breakthrough technology. Many new technologies are initially evaluated using data from randomized controlled trials (RCTs), but as technologies diffuse, they may be applied to different populations by providers with varying skill levels and for indications other than in the original evaluations of the technology's efficacy. Productivity may improve over time because of technical improvements in materials and greater proficiency in provision given a higher volume of service per provider and more years of provider experience with the technology. However, productivity may decline if the technology is increasingly supplied by less proficient providers and/or the technology is applied to patient populations for whom the technologies are less appropriate.

Productivity measurement requires that output is defined, and defining output in the health sector has been particularly challenging. Such measures as the quantity of service may be appropriate for gauging productivity change attributable to process innovations—innovations which lead to increased output per input, but process innovations are relatively less common in health care since financial incentives for such innovations have been lacking [1]. Because of the data availability and clarity of definition (death is unambiguous), measures related to mortality have been widely used to gauge improvements in health status. However, many new health care technologies aim to improve outcomes other than longevity. In such cases, measures of morbidity, pain, and/or physical, mental, or cognitive functioning are more appropriate. Consistently defined time series data are less likely to be available for the latter types of outcome measures.

Osteoarthritis (OA) of the hip and knee are highly prevalent conditions, especially among the elderly [6]. These conditions account for a substantial amount of disability [7, 8] and expenditures on personal health care services, including for hospital inpatient and ambulatory care [9–18]. Viewed from the perspective of Medicare's history, hip and knee replacement have been important innovations, yielding benefits to beneficiaries in improved function and reduction in pain. Yet these procedures are costly to the Medicare program. Studies have documented that these procedures can relieve chronic joint pain and are cost effective [19–22]. Although there is some evidence of underuse [23] and access barriers facing some demographic groups, such as minorities [24, 25], and there are geographic disparities in receipt [26], the procedures are widely diffused. The volume of these procedures has grown and is expected to increase further in future decades [27–29].

This study used 1996–2010 data from the Health and Retirement Study (HRS) on individuals 50 years and older to assess secular trends in productivity of hip and knee surgery measured in terms of changes in physical function after versus before joint surgery over this time period. Hip and knee replacement technology, the principal surgical procedures performed to treat OA of the hip and knee, was mature by the mid-1990s. Hip replacements were first performed in 1940 [30] and knee replacements in 1954 [31], although rapid diffusion of these technologies occurred much later. We use the term “hip and knee surgery” since the HRS did not distinguish between joint replacement and other surgical procedures of the hip and knee in earlier years. Judging from later years' HRS data, which did make this distinction, over nine-tenths of hip surgery and about three-quarters of knee surgery involved a joint replacement.

Except for the failure to identify joint replacement procedures from other forms of hip and knee surgery in the early years of the HRS, the data are well suited for this study. Given the high volume of procedures performed, we were able to monitor changes in physical function in a longitudinal nationally representative sample of individuals aged 50 and over. Fortunately, physical functioning and pain have been measured consistently over time.

## 2. Materials and Methods

### 2.1. The Health and Retirement Study

The HRS is a longitudinal survey of persons aged 51–61 in 1992 and their spouses or partners who could be of any age [32]. Older and younger cohorts were added subsequently [32]. The HRS is conducted in even-numbered years. Presently, 26,000 Americans are surveyed. The HRS is a general survey of labor force participation and the health transitions that individuals undergo toward the end of their work lives and in the years that follow. The HRS collects data on a broad range of variables, including income, work, assets, pension plans, health insurance, disability, physical health and functioning, cognitive functioning, and health care expenditures.

In 1998, HRS was combined with a survey of persons 70+ in 1993, the Aging and Health Dynamics of the Oldest Old (AHEAD). To achieve consistency in question framing, we limited our analysis to 1996–2010.

### 2.2. Treatment and Control Groups

The treatment groups for hip and knee surgery consisted of persons who indicated that they had “surgery or any joint replacement because of arthritis.” A follow-up question was asked for the joint type. For controls, we selected persons who were told by a physician that they had arthritis, took medication for this condition, and were limited in their activities because of arthritis. The 1996 interviews were used exclusively for presurgery (actual or counterfactual) values; 2010 interview data were only used for follow-up values. The surgical procedures for our study were reported in the 1998–2010 interviews. Responses to questions about physical functioning and pain were taken from the same interview at which the surgical procedure was reported for the treatment groups. For the control groups, the comparison was between physical functioning and pain in adjacent HRS waves. The gap between presurgery and postsurgery dates was 2 years. The HRS provided data on health and functional status, demographic characteristics, and family income.

### 2.3. Measuring Physical Functioning

We assessed 6 physical functioning scales. Individual items comprising each scale were coded 1 if the respondent reported having at least some difficulty (including “little difficulty,” “alot of difficulty,” “cannot do,” and “do not do”) in performing the activity. Responses were coded 0 if the person had no difficulty performing the task. These binary values were then summed to yield the individual's value for the scale. Internal consistency and measurement properties, including reliability of the HRS physical functioning variables have been examined previously [33].

The first physical functioning scale, number of Activity of Daily Living (ADL) limitations, included walking across room, dressing oneself, using toilet, bathing or showering, and getting out of bed. Second, number of Instrumental Activity of Daily Living (IADL) limitations included using telephone, managing money, taking medications, shopping for groceries, and preparing hot meals. Third, the mobility limitations index consisted of walking several blocks, walking 1 block, walking across room, climbing several flights of stairs, and climbing 1 flight. Fourth, the large muscle index consisted of sitting for 2 hours, getting up from chair, stooping, kneeling or crouching, and pushing/pulling large object. Fifth, the gross motor index included walking 1 block, walking across a room, climbing 1 flight of stairs, getting out of bed, and bathing or showering. Sixth, the fine motor index included picking up a dime from the floor, eating; and dressing.

Individual items of the indexes overlapped somewhat, particularly for the gross motor and mobility measures. Ability to perform some tasks is at most indirectly related to functioning of joints, for example, managing money, using the telephone, sitting, and eating. However, there may be indirect effects. Greater physical activity made possible by decreased pain and improved functioning of the hip or knee may decrease medication use and increase physical activity and socializing and improve affect, thereby improving the person's ability to perform such routine tasks.

### 2.4. Measuring Pain

The HRS asked, “Are you often troubled with pain?” If the answer was “yes,” we set a binary variable for pain equal to 1. The HRS also asked, “Does the pain make it difficult for you to do your usual activities such as household chores or work?” We defined a second binary variable for pain, which was set to 1 if the person indicated that pain limited activity and was 0 for respondents reporting pain but without accompanying activity limitations or reporting no pain. Third, the HRS asked persons to report whether their pain was “mild,” “moderate,” or “severe.” We defined binary variables for “severe pain,” and for “moderate pain,” with “mild” with no pain, the omitted reference group.

### 2.5. Statistical Approach

To define appropriate control groups for the treatment groups, we used propensity score matching (PSM). The goal of matching on propensity scores is to make the treatment and control groups similar and thereby reduce selection bias in assigning persons to an intervention [34, 35].

Our application of PSM involved 3 steps. First, we performed logit analysis to predict the probability that a beneficiary with the study OA diagnoses received hip or knee surgery. Second, using the predicted probability, we matched a beneficiary who underwent surgery to his/her nearest match among controls. Third, we computed average treatment effects on the treated (ATTs), which measured differences in physical functioning and pain following surgery between the treatment and control groups.

We used nearest neighbor matching and a caliper of 0.02 with PSMATCH2 from Stata 12 (StataCorp. LP, College Station, TX). Observation pairs were dropped if differences in values exceeded this amount.

The quality of the match between treatment and control groups may be considered adequate or inadequate, depending on the magnitude of the standardized difference between the values for the treatment and control groups on each covariate used for matching. A general criterion for adequate matching is that standardized differences for the covariates used in the logit analysis and for matching do not exceed 10% [36–38]. PSMATCH2 gives ATTs and standard errors of the ATTs from which statistical significance was calculated.

Persons with specific demographic characteristics, for example, male gender and higher educational attainment measured in terms of years of schooling completed, and with higher household income are more likely to undergo these surgical procedures. We hypothesized that persons with poorer physical function and with more pain would be more likely to be surgically treated, but fair or poor general health may reduce the probability of receiving these procedures. There may be technological change and changes in criteria for selecting patients for surgery. Thus, it was important to account for year of surgery.

We performed PSM using these covariates defined for the baseline year (HRS interview immediately prior to the report of surgery or the reference date for the control): demographic characteristics—age, race/ethnicity (black, Hispanic, and other races with white race omitted), female gender, married versus not currently married, years of schooling completed (12, 13–15, 16+, <12 omitted), obese-body mass index (BMI) ≥30, fair or poor health from a 5-point scale of self-rated health, the study's physical functioning measures at baseline for the treatment group and before the “reference date” (our terminology for the interview year corresponding to the postsurgery date for the treatment group), the respondent's earnings in the year before baseline, and other household income in the same year. We also included binary variables for whether or not the person was employed at baseline or at the interview before the reference date, and for whether or not the person had health insurance of any type then. Finally, we included a continuous variable for year of interview, defined for the baseline/reference date. The variable ranged from 1 for 1996 and 2 for 1998 to 8 for 2010.

To evaluate productivity change separately for hip and knee surgery, we conducted PSM separately with data from (1) 1998 and 2000, (2) 2002–2006, and (3) 2008–2010 interviews. To gauge productivity trends, we examined trends in ATTs and in associated 95% confidence intervals for the 3 time sub-periods. Matching was conducted for the observational period as a whole. The evaluation of productivity trends used the matched sample for 1996–2010, but we computed ATTs for each sub-period depending on when the surgical procedure (and its matched control) occurred.

## 3. Results

The mean ages of persons undergoing hip and knee surgery were nearly 70 and slightly over 68, respectively (Table 1). Persons receiving knee surgery were more likely to be black or Hispanic, 13% and 6%, than were persons receiving hip surgery, 8% and 6%. Sixty-six percent of persons with knee surgery compared to 61% of persons with hip surgery were female, but 70% of persons in both control groups were female. On average, household income was $47,600 for hip and $45,810 for knee surgery patients. Only 25% of hip and 30% of knee surgery patients were employed.

Persons who underwent joint surgery tended to be more impaired in physical function than those who did not undergo surgery, judging from the mean values on physical functioning prior to matching. Fractions of persons experiencing pain were more similar between the surgery and nonsurgery groups, but if anything, pain tended to be more severe for the controls. Likewise, higher proportions of nonsurgical patients reported that they were in fair or poor health than surgical patients prior to surgery. Surgical patients were more likely to be male, more highly educated, more affluent, and slightly more likely to have had health insurance. Overall, patterns for hip and knee surgeries tended to be similar except for the fraction of persons who were obese at baseline. Hip surgery recipients were slightly less likely to have been obese prior to surgery than controls. Among knee surgery recipients, nearly half of recipients were obese compared to 37% of controls.

For the hip analysis, there were 622 observations before matching. After matching, there were 611 paired treatment/control observations; 11 observations were dropped because an adequate match satisfying our matching criteria could not be found. For the knee analysis, we started with 2,038 observations in the treatment group, which decreased to 2,003 after matching.

Standardized differences between covariates in the treatment versus control groups were substantially reduced after matching (Table 2). The samples were very well balanced between treatment and control groups. The largest standardized difference in the hip surgery analysis was −6.36%, for earnings. For knee surgery, the largest standardized difference was for insurance prior to surgery, −2.73%. Treatment and control groups were well matched on pain measures obtained by the HRS.

Persons surgically treated for arthritis of the hip and knee experienced improved physical function and pain relief over time (Table 3). The ATTs for the observational period as a whole indicate improvements in all physical function and one pain measure for knee surgery and for the majority of measures for hip surgery. Exceptions for hip surgery are mobility, the fine motor index for which improvement would be expected to occur at most indirectly, and pain. For knee surgery, pain was also an exception. For both procedures, however, there were improvements in pain restricting activity. The largest improvements were reductions in ADL limitations for hip surgery and in IADL limitations for both hip and knee surgery.

The results for pain are particularly striking. The ATTs of −0.23 and −0.19 for hip and knee replacement, respectively, for all-year periods compared to pre-surgery proportions with pain of 0.31 and 0.34 for hip and knee surgery, respectively (from Table 2). The ATTs of −0.24 and −0.20 for pain restricting activity are relative to pre-surgery proportions of 0.26 and 0.28. Thus, it appears that receipt of these procedures tended to virtually eliminate patient reports of pain. The lack of trend in pain alleviation attributable to surgery may reflect the large effects on pain reduction already realized by surgical patients in the first subperiod. Effects on physical function are also substantial. For example, the ATTs for mobility limitations are −0.34 and −0.46, which compare to presurgery mean values of 1.97 and 1.94.

For there to be statistically significant differences in ATTs, there can be no overlap in 95% confidence intervals. Based on this criterion, there were no statistically significant productivity improvements despite trends in this direction. Further, differences in some indicators are small, for example, large muscle index outcome for knee surgery with ATTs of −0.22, −0.20, and −0.24 for the 3 subperiods. Some improvements over time, such as for large muscle index for knee surgery, are not monotonic.

## 4. Discussion

This study's empirical evidence indicates that hip and knee surgical procedures for osteoarthritis are effective in improving physical function and decreasing pain, particularly the latter, and with the exceptions already noted, the productivity of these surgical interventions improved over time. These improvements occurred during a period of substantial growth in volumes of these procedures, but there were no technological breakthroughs in hip or knee surgery during this period.

At least three important policy implications follow from our findings. First, it is important to consider productivity growth when evaluating growth in medical care prices and spending—an implication not unique to joint surgery. For example, after adjusting for quality improvements, the unit cost of treating diabetes mellitus between 1999 and 2009 was about constant [4]. This is not a new point [39], but one often forgotten in public policy discussions and hence worth repeating in view of our findings.

Second, particularly when the volume of a particular service increases, there is concern that some of the utilization increase is unwarranted. Because we could not document the precise reasons for the observed trends in productivity, we cannot know the extent to which the growth in volume includes some growth of volume for which benefit falls short of cost. It is possible that productivity increased in spite of growth in such volume, for example, because providers became more proficient in performing the procedures. Nevertheless, the productivity gains suggest that the growth of such nonbeneficial services was not a dominant factor.

Third, the finding that hip and knee surgery patients had better functional and socioeconomic status prior to surgery than controls is both good and bad news. Healthier and less functionally impaired persons may be better candidates for such surgery—possibly good news. On the other hand, especially since the surgical patients tended to have better functional status than nonsurgical patients, the pattern in functional status prior to surgery could reflect access barriers to care faced by individuals with physical impairments due to osteoarthritis of the hip and knee. Virtually every surgical patient had health insurance, but almost the same percentage of controls had health insurance as well. So to the extent that access barriers existed, it was not primarily due to lack of health insurance.

A major strength of this study is the data on which it was based. The Health and Retirement Study is a nationally representative longitudinal sample of individuals. Questions about physical function and pain were consistent over a period of nearly a decade and a half. The questions were asked of all persons rather than pertaining to patients with a particular procedure or condition. Also, since there may be improvements in function over time for reasons other than receipt of a joint surgery, we constructed control groups. The measures of health outcome we used are highly relevant to joint surgery.

We acknowledge these study limitations. First, we considered affirmative responses to the question about joint surgery during the last 2 years although, in later years, the HRS contained separate questions asking whether the surgical procedure involved joint replacement or some other type of joint surgery. The vast majority of surgical procedures of the hip and knee were reported to have involved a joint replacement, but we could not develop a consistent time series for joint replacement procedures since the HRS did not ask about joint replacement versus other types of joint surgery in earlier years. Second, while improved physical functioning and reductions in pain are important goals of joint replacement and hence valid outcome measures, they do not capture an important aspect of technological change, that is, improvements in durability of devices. Patients with osteoarthritis not only seek the improvements we measured but also want their devices to have longer lives. Although the life of a device can be projected, realized length of device life is only knowable much later. In this sense, however, our estimates of the gains from joint surgery if anything represent lower bounds.

Despite these limitations, the results suggest that the productivity of hip and knee surgery increased from the late 1990s through the first decade of the 21st century as measured by improved physical functioning and decreased pain. Such improvements may be expected to result in improvements in self-care capacity of near elderly and elderly adults, which is supported by the results on Activities of Daily Living and Instrumental Activities of Daily Living limitations.

## Figures and Tables

**Table 1:** Hip and knee surgery before matching.

	Hip			Knee		
	Treatment	Control	S Diff	Treatment	Control	S Diff
ADL limitations	0.65	0.77	−10.38	0.46	0.79	−28.85
IADL limitations	0.32	0.54	−23.39	0.24	0.55	−34.98
Mobility limitations	1.98	2.12	−8.63	1.95	2.13	−11.61
Large muscle index	2.07	2.31	−18.23	2.10	2.31	−16.42
Fine motor index	0.37	0.44	−10.05	0.27	0.45	−26.33
Gross motor index	1.01	1.19	−12.37	0.91	1.20	−21.23
Pain	0.32	0.35	−6.73	0.35	0.35	−1.12
Pain restrict	0.27	0.31	−9.66	0.28	0.31	−6.43
Pain moderate	0.19	0.19	0.47	0.19	0.19	1.20
Pain severe	0.07	0.11	−11.76	0.09	0.11	−4.06
Age	69.82	68.70	12.03	68.19	68.87	−7.14
Black	0.08	0.22	−38.25	0.13	0.21	−23.65
Hispanic	0.04	0.10	−23.63	0.06	0.10	−14.70
Other race	0.01	0.05	−20.98	0.03	0.05	−12.28
Female	0.61	0.70	−17.71	0.66	0.70	−8.14
Married	0.66	0.54	23.92	0.66	0.54	25.14
Education 12 yrs	0.36	0.31	10.10	0.36	0.31	9.93
Education 13–15 yrs	0.24	0.17	16.70	0.21	0.17	9.65
Education 16+ yrs	0.22	0.13	24.77	0.18	0.13	14.88
Fair/poor health	0.30	0.55	−51.31	0.35	0.55	−41.85
Obese	0.33	0.37	−7.95	0.49	0.37	24.20
Earnings	7.80	6.06	6.97	9.48	6.01	13.77
Income	47.60	33.04	20.20	45.81	32.82	17.61
Employed	0.25	0.22	7.07	0.30	0.22	18.47
Insurance	0.99	0.95	23.99	0.98	0.95	18.23
Year	4.08	3.90	8.72	4.17	3.91	13.41

*N*	622	9,841		2038	10007	

**Table 2:** Hip and knee surgery after matching.

	Hip			Knee		
	Treatment	Control	S Diff	Treatment	Control	S Diff
ADL limitations	0.64	0.62	1.89	0.47	0.49	−2.29
IADL limitations	0.32	0.29	3.46	0.24	0.25	−1.49
Mobility limitations	1.97	1.96	0.50	1.94	1.98	−2.08
Large muscle index	2.06	2.04	1.37	2.10	2.11	−0.59
Fine motor index	0.37	0.33	6.15	0.27	0.27	0.62
Gross motor index	1.00	1.00	0.24	0.91	0.94	−2.19
Pain	0.31	0.34	−4.54	0.34	0.33	1.79
Pain restrict	0.26	0.28	−4.77	0.28	0.27	1.79
Pain moderate	0.18	0.20	−3.33	0.19	0.19	0.64
Pain severe	0.07	0.08	−3.65	0.09	0.10	−0.51
Age	69.88	69.51	3.99	68.20	68.31	−1.21
Black	0.08	0.08	−0.60	0.13	0.13	−0.60
Hispanic	0.04	0.05	−2.41	0.06	0.07	−2.24
Other race	0.01	0.01	0.00	0.03	0.02	−0.96
Female	0.61	0.60	3.01	0.66	0.67	−2.11
Married	0.66	0.66	−0.69	0.66	0.66	1.48
Education 12 yrs	0.35	0.36	−0.68	0.36	0.35	1.57
Education 13–15 yrs	0.24	0.23	1.16	0.21	0.21	−2.08
Education 16+ yrs	0.22	0.22	1.58	0.18	0.18	−0.52
Fair/poor health	0.30	0.29	2.87	0.35	0.35	−0.94
Obese	0.34	0.34	0.00	0.49	0.49	−0.50
Earnings	7.85	9.35	−6.36	9.47	9.65	−0.48
Income	47.58	53.56	−5.48	46.02	46.14	−0.11
Employed	0.25	0.27	−3.36	0.30	0.30	−1.09
Insurance	0.99	0.99	4.46	0.98	0.99	−2.73
Year	4.07	4.16	−4.42	4.18	4.17	0.41

*N*	611	611		2003	2003	

**Table 3:** Average treatment effects on the treated (ATT) for 1998–2010 and subperiods.

Outcome levels	Hip				Knee			
	Observations	ATT	95% Confidence interval		Observations	ATT	95% Confidence interval	
*All-year periods *								
ADL limitations	1,220	−0.10	(−0.23, 0.04)		4,004	−0.21	(−0.28, −0.14)	∗
IADL limitations	1,220	−0.14	(−0.26, −0.03)	∗	4,004	−0.14	(−0.20, −0.09)	∗
Mobility limitations	1,220	−0.34	(−0.52, −0.16)	∗	4,006	−0.46	(−0.56, −0.36)	∗
Large muscle index	1,220	−0.24	(−0.38, −0.10)	∗	4,004	−0.24	(−0.31, −0.16)	∗
Fine motor index	1,220	0.02	(−0.06, 0.10)		4,004	−0.10	(−0.14, −0.06)	∗
Gross motor index	1,220	−0.33	(−0.49, −0.17)	∗	4,004	−0.33	(−0.41, −0.24)	∗
Pain	1,222	−0.23	(−0.28, −0.18)	∗	4,004	−0.19	(−0.21, −0.16)	∗
Pain restrict	1,222	−0.24	(−0.29, −0.18)	∗	4,004	−0.20	(−0.23, −0.17)	∗

*Subperiod results *								
ADL limitations								
1998–2000	340	0.07	(−0.21, 0.35)		958	−0.14	(−0.29, 0.01)	
2002–2006	496	−0.11	(−0.31, 0.09)		1,812	−0.15	(−0.26, −0.05)	∗
2008–2010	382	−0.17	(−0.41, 0.07)		1,228	−0.21	(−0.33, −0.09)	∗
IADL limitations								
1998–2000	340	−0.01	(−0.24, 0.21)		958	−0.05	(−0.16, 0.07)	
2002–2006	496	−0.12	(−0.28, 0.03)		1,812	−0.10	(−0.19, −0.02)	∗
2008–2010	382	−0.25	(−0.47, −0.03)	∗	1,228	−0.17	(−0.27, −0.07)	∗
Mobility limitations								
1998–2000	340	−0.69	(−1.04, −0.34)	∗	958	−0.43	(−0.64, −0.22)	∗
2002–2006	496	−0.43	(−0.71, −0.15)	∗	1,812	−0.52	(−0.67, −0.37)	∗
2008–2010	382	−0.50	(−0.83, −0.17)	∗	1,228	−0.48	(−0.66, −0.31)	∗
Large muscle index								
1998–2000	340	−0.21	(−0.47, 0.04)		958	−0.22	(−0.37, −0.07)	∗
2002–2006	496	−0.39	(−0.60, −0.17)	∗	1,812	−0.20	(−0.31, −0.08)	∗
2008–2010	382	−0.46	(−0.71, −0.21)	∗	1,228	−0.24	(−0.37, −0.10)	∗
Fine motor index								
1998–2000	340	0.13	(−0.03, 0.29)		958	−0.05	(−0.14, 0.04)	
2002–2006	496	−0.05	(−0.18, 0.07)		1,812	−0.09	(−0.15, −0.03)	∗
2008–2010	382	−0.13	(−0.27, 0.01)		1,228	−0.10	(−0.17, −0.03)	∗
Gross motor index								
1998–2000	340	−0.14	(−0.46, 0.18)		958	−0.27	(−0.45, −0.09)	∗
2002–2006	496	−0.27	(−0.51, −0.04)	∗	1,812	−0.31	(−0.44, −0.19)	∗
2008–2010	382	−0.40	(−0.68, −0.12)	∗	1,228	−0.34	(−0.49, −0.19)	∗
Pain								
1998–2000	340	−0.26	(−0.36, −0.16)	∗	958	−0.17	(−0.23, −0.11)	∗
2002–2006	496	−0.25	(−0.33, −0.17)	∗	1,812	−0.19	(−0.23, −0.15)	∗
2008–2010	382	−0.25	(−0.34, −0.16)	∗	1,228	−0.17	(−0.23, −0.12)	∗
Pain restrict								
1998–2000	340	−0.22	(−0.32, −0.11)	∗	958	−0.14	(−0.20, −0.08)	∗
2002–2006	496	−0.24	(−0.32, −0.15)	∗	1,812	−0.20	(−0.25, −0.16)	∗
2008–2010	382	−0.31	(−0.41, −0.22)	∗	1,228	−0.20	(−0.25, −0.14)	∗

*Statistically significant < 0.05.
